# Probe Lasso: A novel method to rope in differentially methylated regions with 450K DNA methylation data

**DOI:** 10.1016/j.ymeth.2014.10.036

**Published:** 2015-01-15

**Authors:** Lee M. Butcher, Stephan Beck

**Affiliations:** UCL Cancer Institute, University College London, London WC1E 6BT, UK

**Keywords:** Differentially methylated regions, DNA methylation, Epigenetics, EWAS, Illumina 450K BeadChip

## Abstract

The speed and resolution at which we can scour the genome for DNA methylation changes has improved immeasurably in the last 10 years and the advent of the Illumina 450K BeadChip has made epigenome-wide association studies (EWAS) a reality. The resulting datasets are conveniently formatted to allow easy alignment of significant hits to genes and genetic features, however; methods that parse significant hits into discreet differentially methylated regions (DMRs) remain a challenge to implement. In this paper we present details of a novel DMR caller, the Probe Lasso: a flexible window based approach that gathers neighbouring significant-signals to define clear DMR boundaries for subsequent in-depth analysis. The method is implemented in the *R* package *ChAMP* (Morris et al., 2014) and returns sets of DMRs according to user-tuned levels of probe filtering (e.g., inclusion of sex chromosomes, polymorphisms) and probe-lasso size distribution. Using a sub-sample of colon cancer- and healthy colon-samples from TCGA we show that Probe Lasso shifts DMR calling away from just probe-dense regions, and calls a range of DMR sizes ranging from tens-of-bases to tens-of-kilobases in scale. Moreover, using TCGA data we show that Probe Lasso leverages more information from the array and highlights a potential role of hypomethylated transcription factor binding motifs not discoverable using a basic, fixed-window approach.

## 1 Introduction

DNA methylation is an essential epigenetic modification for normal mammalian development. It refers to the addition of a methyl group at the 5′ position of cytosine nucleotides (C) to form 5-methylcytosine (mC) and in mammalian cells occurs predominantly at CpG dinucleotides. CpG dinucleotides are underrepresented in mammalian genomes but the majority of these loci (70–80%) in a given cell population exhibit high levels of methylation (mC_locus_: >85%). CpGs that remain constitutively unmethylated tend to cluster into CpG-rich regions called CpG islands (CGIs). Curiously, pluripotent stem cells harbour an additional 33% of mC at non-CG (CpH) dinucleotides, however; this epigenetic mark is less stable and, consequently, these loci often exist as partially methylated (mC_locus_: 25–50%). Although in theory every methylated cytosine has the potential to become de-methylated, less than 22% of autosomal CpGs are dynamically regulated [2]. Nevertheless, the prevailing pattern of variation in DNA methylation leaves a cell-specific stamp, which together with other epigenetic alterations such as histone modifications and non-coding RNAs, contribute to a series of exquisitely coordinated mechanisms that control gene expression both temporarily and spatially.

Correct acquisition of DNA methylation in proliferating cells is governed by the DNA methyltransferases (DNMT), a family of three catalytically-active enzymes comprising maintenance (DNMT1) and *de novo* (DNMT3a and DNMT3b) functions. Perturbation of these genes in mouse results in a range of detrimental phenotypes, which highlights the indispensable role of DNA methylation in normal development. These phenotypes include genome-wide partial methylation loss, developmental delay, erroneous germline imprints, sterility and embryonic lethality.

Given the clear importance of DNA methylation, concerted efforts are underway to understand the impact of more subtle DNA methylation differences on normal development and disease. Our understanding is gradually coming into focus due to a number of high-information content methylation technologies that have emerged in the last 5 years (reviewed in [3]). These include whole genome bisulfite sequencing (WGBS; [4], [5]), methylated immunoprecipitation sequencing (MeDIP-seq; [6]), reduced-representation bisulfite sequencing (RRBS; [7]) and the Illumina Infinium Human Methylation 450K BeadChip (herein termed, “450K BeadChip”; [8]). All these platforms are capable of generating genome-wide or whole genome methylation profiles (“methylomes”) and deliver high-information content, albeit with different foci [9]. For example, although WGBS is not amenable to studying large cohorts (due to the number of reads required to cover each cytosine with sufficient depth), it can resolve entire methylomes at single nucleotide-resolution; on the other hand, 450K BeadChips only assay approx. 1.8% of CpGs but are highly amenable to studying large cohorts – a critical requirement for statistical power; MeDIP and RRBS lie somewhere in between. All the aforementioned technologies with the exception of MeDIP-seq, use bisulfite converted DNA to resolve mC at single base-resolution; in contrast, MeDIP uses an antibody to enrich for the methylated fraction of the genome and provides a region-based “consensus” methylation level, with resolution concomitant with sequence insert size [10].

Because sequence-based approaches often provide broad and uninterrupted methylomic coverage it is not surprising that these technologies are responsible for identifying the bulk of differentially methylated regions (DMRs) that distinguish cell-, tissue- and disease-specific phenotypes. DMRs are discrete genomic sequences that harbour a distinct methylation signature across a number of CpGs (and/or non-CpGs) capable of distinguishing one phenotype from another. Their identification and utility have far-reaching implications for clinical applications because they ultimately reduce the scale of the genome to a handful of regions; once DMRs are validated and replicated it paves the way for time-, cost- and effort-effective assays that will inform subsequent functional studies and deliver diagnostic tools.

Even though the majority of DMRs have been identified using sequencing-based methods, the majority of methylomes have been generated using the 450K BeadChip; for example, the latest version of the MARMAL-AID database [11] contains 450K data for more than 9000 samples from nearly 200 different tissues and almost 100 different diseases. For technical manufacturing reasons though, the coverage of CpGs on 450K BeadChips has to be restricted. As a result, and possibly for historical reasons, CpG distribution on the 450K BeadChip is skewed towards CGIs and genes. Moreover contiguous CpGs are not always covered. This has therefore opened up the challenge to implement a comprehensive algorithm for DMR calling on 450K BeadChip datasets. A simple approach is to count significant signals emanating from a fixed-size sliding-window. This way a DMR could be defined if a window (or contiguous windows) of certain size capture a specified number of significantly associated probes. As discussed above however, this is contentious due to the distribution of CpGs and risks restricting DMR calling to regions most heavily probed. There are a number of DMR calling methods within the public domain that have application to the 450K BeadChip. These include ‘Bump Hunting’ [12], ‘Block Finding’ [13], ‘AClust’ [14], and ‘DMRcate’ [15].

Here we introduce an alternative DMR calling method, the Probe Lasso. Probe Lasso utilises a flexible window (“probe-lasso”) based on probe density to gather neighbouring significant-signals to define clear DMR boundaries. The principal motive for developing this algorithm is to redirect subsequent analysis away from just probes/regions located in promoters/CGIs, which the array is skewed toward and leverage information from putatively important, but largely ignored, intergenic regions. To illustrate this we benchmark Probe Lasso against a fixed window approach. Probe Lasso shares similarities with another DMR calling method, ‘Comb-p’ [16] although there are notable differences; in particular, Comb-p uses auto-correlation data to first correct individual probe *p*-values, then defines DMRs based on peaks of corrected *p*-values. In contrast, Probe Lasso gathers neighboring significant signals from probes in regions that can extend according to the probe’s genomic/epigenomic annotation and then uses auto-correlation information to combine the *p*-values of probes within a DMR.

## 2 Materials and methods

### 2.1 Preprocessing and methylation-variable position (MVP) calling

Probe Lasso is implemented within the Bioconductor package *ChAMP* [1], and relies on a series of objects created using this package. The following provides a brief description of a typical workflow using *ChAMP*. Raw data (.idat files) are loaded using the champ.load function to derive a list object that contains, among other things, methylation levels (‘beta’) of probes for samples specified in a sample sheet (‘pd’) and detection *p*-values (‘detP’) for each probe. We remove samples with call rates (i.e., detP <0.01) less than 98%, and then remove probes that do not provide complete information across all samples. Beta values are inter-array normalised using one of a variety of publically available procedures with the champ.norm function and subject to singular variable decomposition (SVD) analysis with champ.svd to identify potential confounding factors. MVPs are then identified for appropriate contrasts using champ.mvp, which implements the *limma* package [17] and the resulting object is used for DMR calling using champ.lasso.

### 2.2 Dependencies

To call DMRs effectively champ.lasso requires each probe to have genetic and epigenetic feature annotation and polymorphism data. Genetic and epigenetic feature annotation is maintained in the Bioconductor package *IlluminaHumanMethylation450kmanifest* and contains information such as chromosome, mapping position, nearby genes and/or CGIs; polymorphism data is held in the Bioconductor package *Illumina450ProbeVariants.db*, which contains allele frequency information of variants within a probe, within 10 bp of target locus or at target locus for four different ancestry groups (African, American, Asian and European) derived from 1000 Genomes Project [18] data.

### 2.3 Probe Lasso rationale

**Fig. 1:**
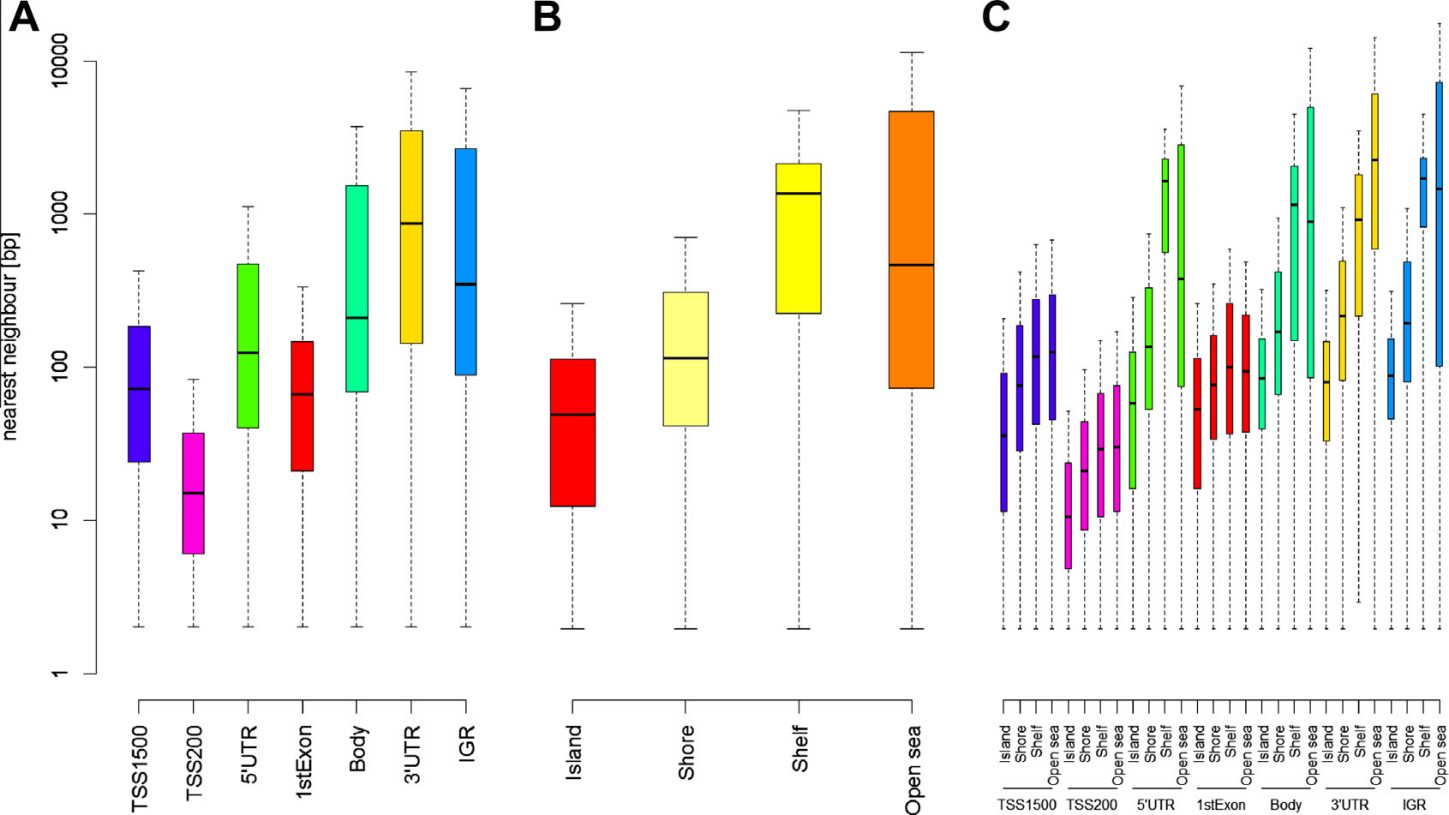
Probe spacing on the Illumina 450K BeadChip. (A) Probes are gene-centric, with those near transcription start sites (TSSs) most densely spaced. (B) Probe spacing is sparser the further a probe’s distance from a CpG island (CGI). (C) Combining genetic and epigenetic annotation information reveals a diverse range of probe spacing.

Fig. 1A illustrates that probe spacing on the 450K BeadChip is not uniform with regard to gene feature: probes within 200 bp of a transcription start site (“TSS200”) are most densely spaced whereas probes in 3′ UTRs and intergenic regions (“IGRs”) are least-densely spaced. Unsurprisingly, given the definition of CGIs and their derivatives [8], Fig. 1B reveals that probe density decreases the further a probe maps from a CGI (CGI → shore → shelf → open sea). Furthermore, probe spacing at a specific gene feature covaries with its CGI relation (herein termed “genetic/epigenetic feature”), which diversifies probe spacing even more (Fig. 1C). Taken together, these figures show that gathering neighbouring significant signals on the 450K BeadChip requires a dynamic calling framework.

### 2.4 Probe Lasso workflow

**Fig. 2:**
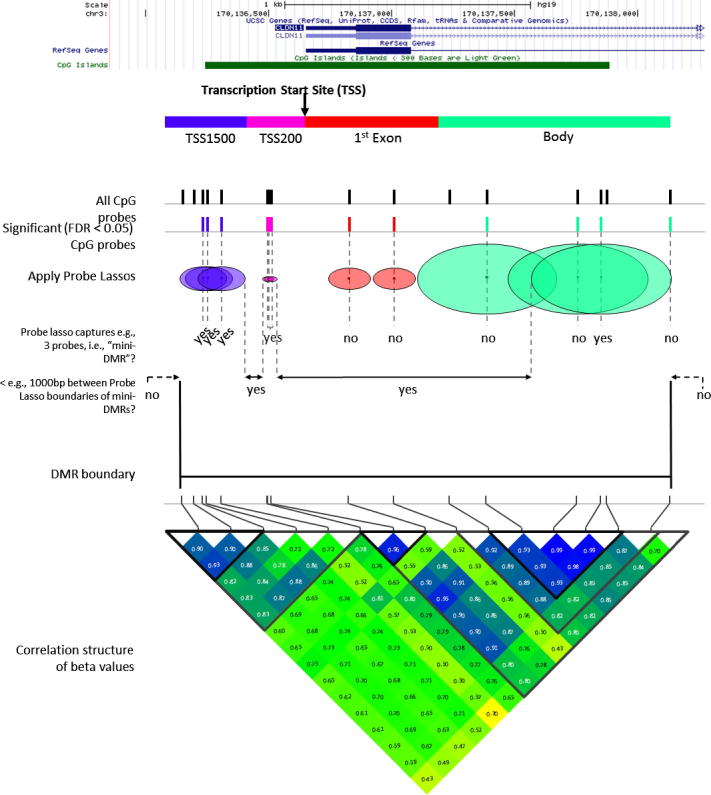
Schematic figure illustrating the Probe Lasso workflow. After probe spacing distributions have been calculated for each of the 28 genetic/epigenetic features, a quantile is set that is based on a user-specified min/max lasso size and lasso radius. This quantile results in 28 dynamic window sizes (‘probe-lassos’) that are thrown around each significantly-associated probe. If these lassos capture a user-specified number of significant probes, that probe’s lasso boundaries are retained. Overlapping- and neighbouring-lasso boundaries less than a user-specified distance apart are then merged to define DMR boundaries. All probes in the dataset are then binned into the DMRs and their *p*-values combined for the DMR, weighted by the underlying correlation structure of probe methylation values.

To account for uneven probe spacing, Probe Lasso generates dynamic, flexible windows (“lassos”) that are tailored to local feature content. Fig. 2 summarises how Probe Lasso calls DMRs. Much like the real thing, probe-lassos can be envisaged as having a centre and a radius; once derived, a probe-lasso is “thrown” around a probe and its radius extends upstream and downstream, centred on the targeted CpG itself. (Meaningful DMR calling using non-CG loci exclusively cannot be facilitated owing to their scant distribution on the 450K BeadChip.) Importantly, probe-lasso derivation is entirely dataset- and user-specific. For example, datasets can be filtered *a priori* for probes mapping to sex chromosomes (filterXY); datasets can also be filtered for the inclusion/exclusion of probes potentially affected by polymorphisms of a specified minor allele-frequency (mafPol.lower, mafPol.upper) in a chosen population (popPol); also, only probes with association statistics inform probe spacing calculations to derive probe-lassos.

**Fig. 3:**
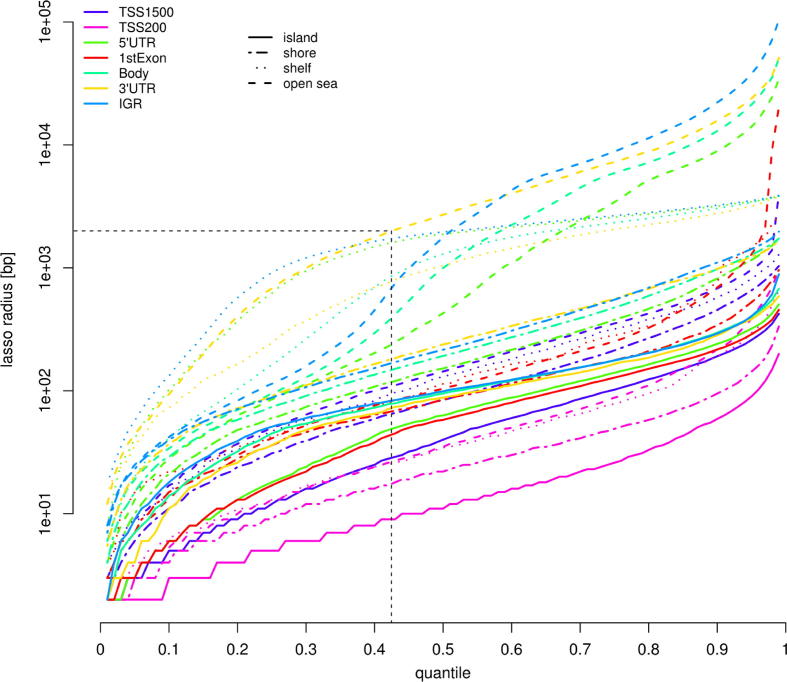
An example quantile distribution of probe spacing for each gene/CGI feature. The black horizontal and vertical dashed lines indicate the quantile (43rd) that results from choosing a maximum lasso size of 2000 bp.

Following probe filtering, Probe Lasso calculates probe spacing for each probe in the dataset; these data are binned into one of the 28 genetic/epigenetic categories (i.e., 7 gene features × 4 CGI relations) and transformed into quantile distributions. Next, a contingency is set up depending on two user-specified parameters, lassoStyle and lassoRadius. If lassoStyle = max, the probe-lasso sizes will be at most 2 × lassoRadius bp; if lassoStyle = min, the probe-lassos will be at least 2 × lassoRadius bp. Because each genetic/epigenetic category has unique probe spacing, Probe Lasso identifies the genetic/epigenetic category that conforms to user-specified maximum (or minimum) lassoRadius and derives the quantile at which it occurs. The derived quantile is then applied to each genetic/epigenetic distribution of probe spacings to create probe-lassos that vary according to genetic/epigenetic-feature (see Fig. 3).

Depending on which genetic/epigenetic feature a probe maps to, an appropriately sized probe-lasso is thrown around each probe, centred at the target locus. Probe Lasso counts the number of significant probes caught within the probe-lasso bounds and a probe is selected if this number is greater than or equal to the user-specified threshold, minSigProbesLasso. Champ.lasso produces a map of probe-lasso boundaries so that overlapping- and neighbouring lassos are merged if they are separated by less than the user-specified threshold, minDmrSep. A DMR is called when probe-lasso boundaries cease merging. DMR coordinates are defined by the minimum and maximum genomic coordinates of probe-lasso boundaries for probes in the DMR. A secondary set of coordinates are also output, known as the ‘DMR core’, defined by the minimum and maximum genomic coordinates of probes within the DMR. DMRs that are smaller than the user-specified parameter, minDMRsize are filtered out from subsequent analysis.

Next, a *p*-value is estimated for the DMR itself. Because DNA methylation levels at neighbouring probes can be substantially correlated [19], Fisher’s method for combining *p*-values is inappropriate. Instead, Probe Lasso uses Stouffer’s method [20] to assign weights to individual *p*-values, which are based on the underlying correlation structure of measured beta values, before combining them. To this end, Probe Lasso recovers all normalised beta values (from champ.norm) and *p*-values (from champ.MVP) of probes in the dataset captured in a DMR. A correlation matrix of normalised beta values within each DMR is calculated, which is then used to weight each probe’s *p*-value by the inverse sum of its squared correlation coefficient. This has the effect of down-weighting *p*-values of highly correlated (non-independent) probes and up-weighting *p*-values of uncorrelated (independent) probes. *p*-Values for DMRs are corrected for multiple testing with the false discovery rate (FDR) method [21].

In the end, Probe Lasso returns a data frame containing all probes in all DMRs, along with genome annotation for each probe and DMR details such as DMR coordinates, size and FDR-corrected *p*-value.

### 2.5 Probe Lasso and sliding fixed-window parameters

As a proof-of-principle we benchmarked the Probe Lasso algorithm against a sliding fixed-window approach to DMR calling using data from The Cancer Genome Atlas (TGCA, http://cancergenome.nih.gov; [22]). To ensure the MVP list was consistent between algorithms, we kept the following probe-filtering parameters constant across both algorithms: filterXY = TRUE, mafPol.lower = 0, mafPol.upper = 0, popPol = “eur”. The following DMR-classifiers were kept constant across both algorithms: minDmrSep = 1000, and minDmrSize = 0. We also set the threshold of significance for MVPs captured by lassos/windows using adjPVal = 0.05. Finally, the algorithms were compared for increasingly stringent DMR-calling conditions, by varying the minimum number of significant probes captured in a lasso/window to 3, 5 and 7 (i.e., Probe Lasso algorithm: minSigProbesLasso = 3|5|7). Parameters exclusive to the Probe-Lasso algorithm that controlled the dynamic nature of probe-lasso sizes were set as follows: lassoStyle = “max”, lassoRadius = 2000.

For the sliding fixed-window approach we chose three window sizes: 250 bp, because this returned a comparable number of probes/DMRs when using Probe Lasso with the above mentioned parameters; 750 bp, because this corresponded to the mean size of dynamic windows using Probe Lasso with the above mentioned parameters; and an extreme case of 2000 bp. Contiguous windows overlapped 50%. Sliding fixed-window approaches are referred to herein as ‘window.250’, ‘window.750’ and ‘window.2000’.

## 3 Results and discussion

### 3.1 Dataset

We downloaded raw intensity data (idat files) for 38 normal colon and 40 colorectal cancer samples from TCGA and fed these through the ChAMP pipeline. After filtering out one normal colon sample that had <98% of probes passing detection filters (*p* < 0.01), we filtered out probes mapping to the sex chromosomes, non-CG probes and probes with at least 1 poor-detection value (*N* = 22,720). The final dataset comprised 448,832 autosomal probes in 77 samples. We found no evidence of technical confounders using champ.SVD (see Supplementary Fig. 1).

### 3.2 Methylated variable positions and DMR calling

After implementing champ.MVP we found 192,981 MVPs (FDR <0.05) and these were distilled into DMRs using Probe Lasso (‘lasso DMRs’) and a sliding window approach (‘window DMRs’). Implementing Probe Lasso as part of champ.lasso outputs a series of figures that allows the user to gauge whether their chosen parameters were set appropriately. These are: the quantile distribution of probe spacing split by genetic/epigenetic feature (Fig. 3), showing the quantile derived from user specified parameters; a plot showing the sizes of lassos thrown around significant probes (Supplementary Fig. 2); and the dataset-specific range of probe spacing split by genetic/epigenetic feature (Supplementary Fig. 3).

### 3.3 DMR localization

**Fig. 4:**
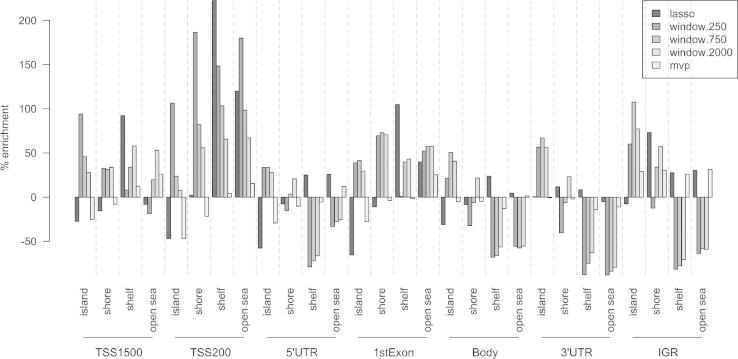
Enrichment plot illustrating the distribution of genetic/epigenetic features for probes captured using the Probe Lasso algorithm (dark-grey bars), a sliding-fixed window approach (mid-greys) and all MVPs (light-grey). As predicted, the sliding fixed window approach enriched for probes near transcription start sites (TSSs) and CGIs. Conversely, the Probe Lasso enriches for CGI shelves and open sea, which is more in keeping with the genetic/epigenetic features of all MVP probes.

The principal aim of the Probe Lasso is to moderate DMR calling away from regions that are simply more probe-dense. We calculated the enrichment of genetic-, epigenetic- and genetic/epigenetic-features for probes in lasso DMRs, window DMRs and for the global MVP distribution. We used previously published definitions of epigenetic relations [8]. Fig. 4 and Supplementary Fig. 4A and B illustrates that window DMRs, regardless of window size, were heavily skewed towards probe dense regions (e.g., most genetic features within CGIs and some CGI shores) and away from probe-sparse regions (e.g., 5′ UTRs, gene bodies, 3′ UTRs and intergenic regions occurring within CGI shelves and open sea). There were significant differences in feature enrichment profiles for all three window-based approaches (with all three stringencies) compared with those of just MVPs (*p*-value range: 0.0015–0.0109, Kolmolgorov–Smirnov test). In contrast, lasso DMR enrichment profiles were not significantly different (*p* > 0.1, Kolmolgorov-Smirnov test) to the enrichment of just MVPs, apart from when 7 probe stringency was employed (*p* = 0.026).

### 3.4 DMR coverage

Table 1 summarises the number of DMRs called using both algorithms for each of the three different stringencies. Comparing like-for-like window sizes (window.750) we can see that the sliding fixed-window approach is less discerning than Probe Lasso across all three stringencies: under the least stringent condition (minSigProbesLasso = 3) nearly a fifth of all probes tested fall into window DMRs. Reducing the window size by two-thirds (window.250) resulted in a similar output of probes and DMRs as Probe Lasso, whereas increasing the window size (window.2000) resulted in little data reduction from the original MVP list, with over three-quarters of significant probes and a third of all probes tested being binned into DMRs. As expected, increasing stringency reduced the number of DMRs called, to the point where less than a 1 Mb of genetic- and epigenetically-diverse sequence can be followed up for targeted analysis (e.g., Probe Lasso, 7 significant probes per lasso).

**Table 1:** Summary of the number of DMRs and probes within DMRs called using the Probe Lasso and fixed sliding-window approach. Sequence overlap is relative to the amount of sequence discovered in Probe Lasso DMRs.

Algorithm	Stringency (# probes)	# DMRs	# Probes	Total DMR sequence [Mb]	DMR sequence overlap [%]	Total DMR core sequence [Mb]	DMR core sequence overlap [%]
Probe Lasso	3	7028	38,524	10.0		4.8	
Window.250		7416	41,028	3.3	15.0	2.2	19.4
Window.750		13,458	92,470	16.7	43.5	10.2	46.2
Window.1000		16,178	148,323	52.3	81.3	28.2	82.9

Probe Lasso	5	1226	11,556	2.6		1.5	
Window.250		1428	11,335	0.5	5.3	0.4	6.7
Window.750		4425	39,455	4.7	22.9	3.2	25.7
Window.2000		7385	84,610	22.2	67.9	13.8	70.2

Probe Lasso	7	395	5035	0.9		0.6	
Window.250		400	4068	0.1	3.7	0.1	4.5
Window.750		1690	18,748	1.7	17.2	1.2	18.4
Window.2000		3840	52,443	11.0	58.6	7.4	61.4

**Fig. 5:**
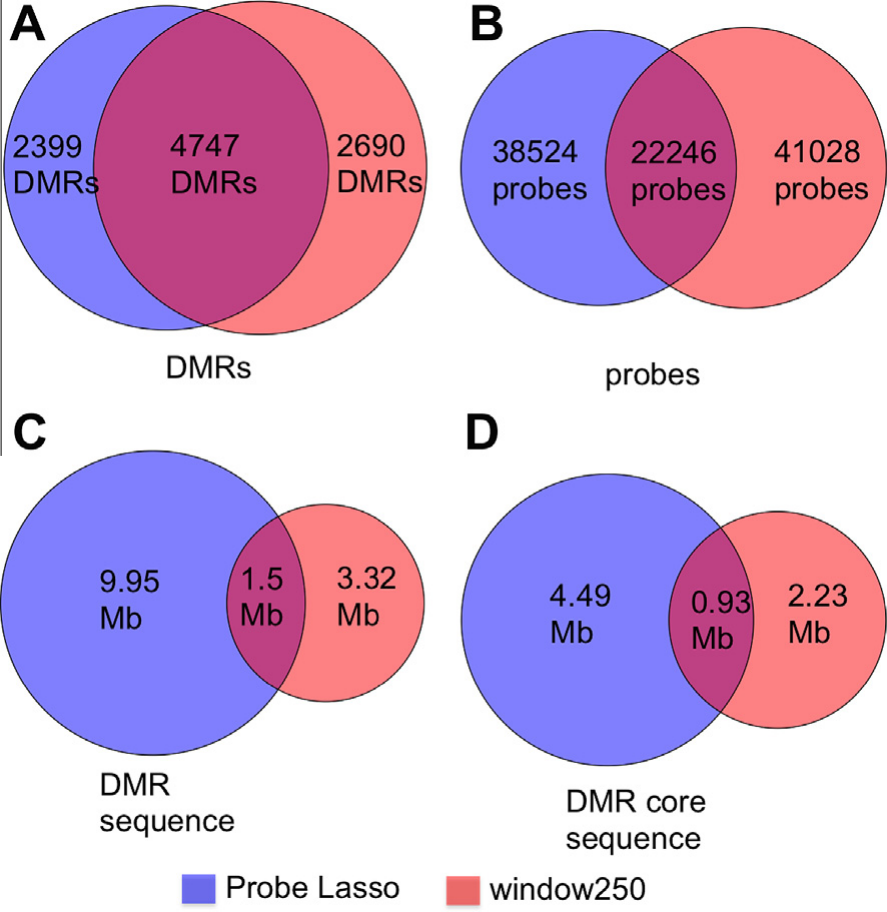
DMR, probe and sequence sharing between Probe Lasso and window.250 algorithms. Approximately 50% of DMRs are shared between the two methods (A), but the number of probes shared is less (B). When DMR sequences are analysed, we see a drastic reduction in shared information (C), which is due to Probe Lasso DMRs leveraging more information from IGRs, which are typified by lower CpG density. This trend is maintained even when probe-lasso boundaries are controlled for (D).

We next assessed the uniqueness of window DMRs and Probe Lasso DMRs. With stringency at its lowest and comparing algorithms with similar numbers of DMRs (window.250), 24.3% of all DMRs were unique to Probe Lasso and 27.3% were unique to window DMRs (Table 1 and Fig. 5A). The number of unique DMRs increased with stringency: 31.6% of lasso DMRs and 38.7% of window DMRs were unique using 5 significant probes; and 37.3% of lasso DMRs and 37.7% of window DMRs were unique using 7 significant probes. The proportion of probes unique to each algorithm was higher than the proportion of unique DMRs (Probe Lasso: 37.8%; window.250: 40.3%; Fig. 5B).

When sequence sharing was analysed we observed even more exclusivity: for example, of the 9.95 Mb of sequence covered by lasso DMRs (using 3 significant probes), 63.7% was unique (Table 1 and Fig. 5C). Moreover, sequence exclusivity for DMRs from different algorithms rose disproportionally to DMR exclusivity when stringency was increased to 5 probes (78.7% unique sequence of 2.6 Mb) and then 7 probes (83.8% unique sequence of 0.9 Mb). To confirm this was not due to “dead space”, that is, DMR boundaries extending into regions for which we have no data, we constrained each DMR boundary to the genomic coordinates of the first and last probe in each DMR (the “DMR core”). Here we observed a similar trend with increased stringency: for 3 probes, 54.9% of the 4.8 Mb DMR core sequence was unique to Probe Lasso DMRs; for 5 probes 75.4% of 1.53 Mb was unique; and for 7 probes 81.5% of 0.62 Mb was unique. So although the two algorithms pick DMRs that overlap by nearly 50%, the sequences likely to be followed up could differ drastically.

**Fig. 6:**
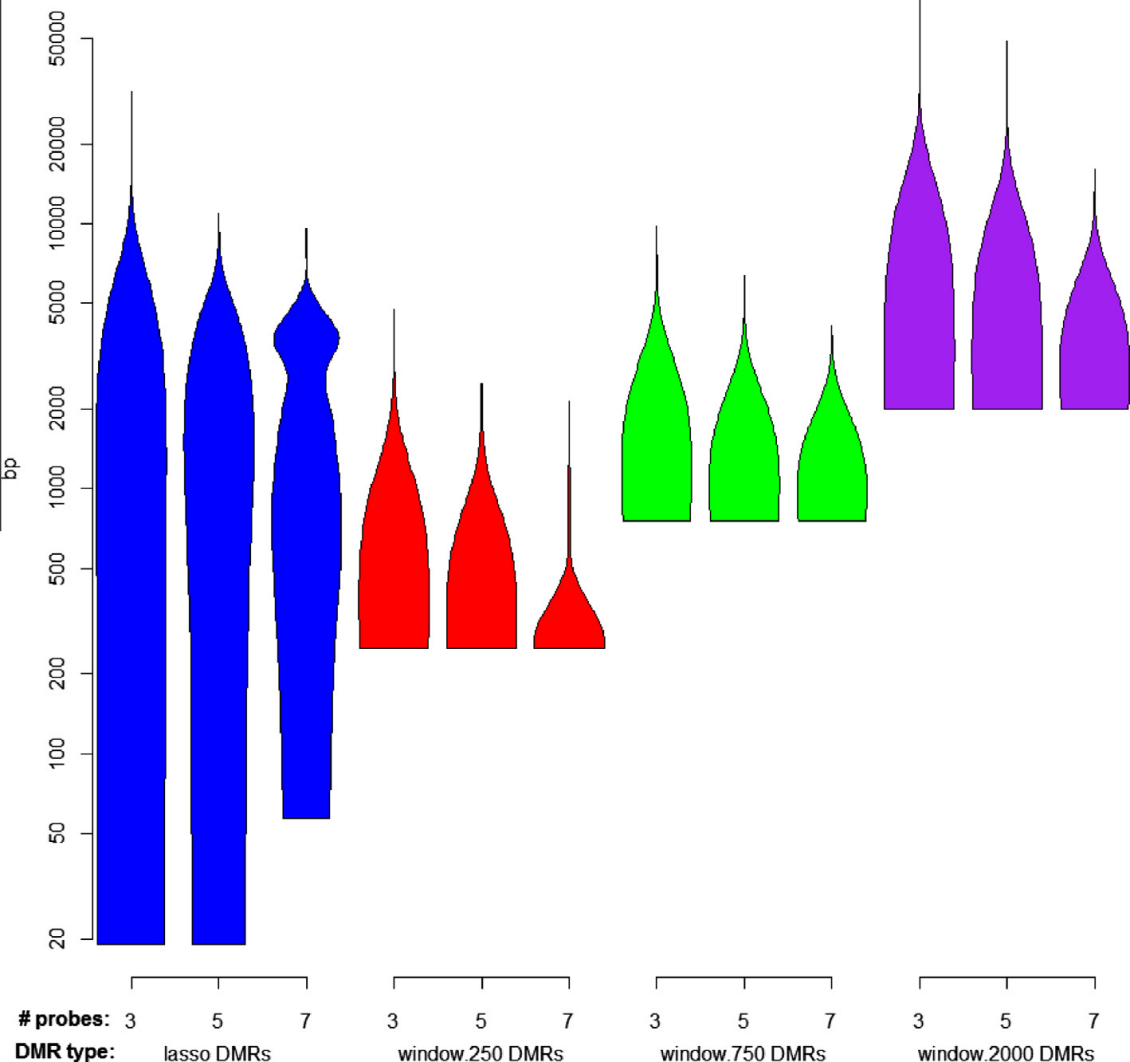
Violin plots demonstrating the distribution of DMR sizes using the Probe Lasso and sliding fixed-window approach with different levels of stringency. Generally, the Probe Lasso captures a wider range of DMR sizes while the smallest DMRs captured by a sliding-window based approach are often constrained to the size of a non-overlapping window. Overall, the Probe Lasso accomplishes a similar job to the combined effort of sliding windows of various sizes, without generating an unwieldy number of DMRs.

This is partly due to Probe Lasso picking DMRs across a range of genetic- and epigenetic-features and is reflected in the distribution of DMR sizes. Fig. 6 shows that Probe Lasso calls DMRs ranging from 19 bp through to 25 Kb. It also highlights that fixed-window approaches are somewhat constrained to calling DMRs only as small as the window size, although this is ameliorated by focussing on DMR cores (Supplementary Fig. 5). Nonetheless, Probe Lasso strikes a decent balance between a wide-range of DMR sizes and a focussed number of DMRs.

### 3.5 Biological relevance of DMRs

Because of the disparity between the number of DMRs defined by window.750 and window.2000 with Probe Lasso, we focussed our attention on comparing Probe Lasso DMRs with window.250 DMRs. Analysis of each DMR set revealed both methods detect hypermethylated DMRs in previously associated colorectal cancer genes such as *BMP3*, *EYA2*, *ALX4* and *VIM* [23] as well as *MLH1* [24]. However, focussing on significant probes in DMRs unique to each algorithm (Probe Lasso: *N* = 8947; window.250: *N* = 11,708) reveals interesting differences between each method. Firstly, and possibly unsurprisingly, significant probes in unique window.250 DMRs were most often found in TSS-associated regions (TSS1500 = 21.5%; TSS200 = 36.7%) and CGIs (71.6%); on the other hand, significant probes in unique Probe Lasso DMRs were most likely to be found in gene bodies (30.3%) and IGRs (48.7%), and highly likely to be in Open Sea (60%). Interestingly, there was little difference in the representation of 5′UTR genomic features (8.0% vs. 6.9%) and CGI shores (20.4% vs 18.5%).

As a means of characterizing the unique DMRs we next sought to identify putative biological processes associated with unique DMRs. We performed a motif analysis using the Discriminative Regular Expression Motif Elicitation (DREME; [25]) tool using 16mers centred around the target sites of significant probes in DMRs unique to each algorithm. The number of motifs discovered in each DMR set was small (window.250 vs. Probe Lasso: 5 vs. 4) but proportional to the number of probe motifs input from each algorithm (11,708 vs. 8947). These motifs were then submitted to Tomtom [26] to identify possible DNA binding proteins. A total of 42 potential DNA binding proteins were associated with the 9 motifs identified in both DMR sets. Of these, nine were in common and included STAT members, EGR2 and N-MYC. There were 11 DNA binding proteins associated with unique Probe Lasso DMRs and these included PAX family members, EHF and PPARG; the remaining 21 proteins were unique to window.250 DMRs and included E2F1-, KLF- and SP-family members (see Supplementary Table 2 for details). It is perhaps not surprising that more motifs were associated with DNA binding proteins in the unique window.250 DMR set given the skew towards probes near transcription start sites, where DNA binding activity is higher; however, it is interesting to note that the motifs discovered in the unique Probe Lasso DMR set were associated with a more diverse range of Gene Ontology predictions, potentially highlighting novel pathways previously ignored due to historical preference for focussing on gene promoters (see Supplementary Table 2).

Finally, we examined the subset of probes that matched significantly-associated motifs to gauge whether unique DMRs could be characterised by unique DNA methylation patterns. Curiously, we found probes mapping to Probe Lasso-derived motifs showed a strong tendency for hypomethylation in colorectal samples (see Supplementary Fig. 6A), possibly leaving these binding sites open to the actions of transcription factors. Conversely, the opposite pattern was found for window.250-derived motifs, suggesting increased affinity for transcription at putative tumor suppressor genes (see Supplementary Fig. 6B).

## 4 Conclusions

In this paper we present the Probe Lasso, a method for calling DMRs using Illumina 450K Methylation BeadChip arrays. Probe Lasso is implemented as part of suite of functions in the Bioconductor package, *ChAMP* – an all-in-one analysis pipeline that takes raw methylation data and derives MVPs and DMRs for further investigation. Probe Lasso has considerable advantages over more basic methods of DMR calling, such as sliding-fixed window approaches. The first is that DMRs are not skewed towards probe-dense regions, and is capable of leveraging more information from the array. Secondly, using a small subset of TCGA data we show that Probe Lasso DMRs highlight the role of hypomethylated transcription factor binding motifs that play key roles in potentially novel pathways. Finally, the Probe Lasso algorithm introduces a framework that could lend itself to DMR calling with whole-genome bisulfite sequencing; here, instead of using probe spacing, and because WGBS benefits from complete coverage, CpG density could be used to mark out DMR boundaries.

## Appendix A Supplementary data

Supplementary data This document contains supplementary figures and tables.
