# Supplementary material for: Probe Lasso: A novel method to rope in differentially methylated regions with 450K DNA methylation data
Source: Methods. 2015 Jan 15;72:21–8. doi: 10.1016/j.ymeth.2014.10.036 (PMC4304833; doi:10.1016/j.ymeth.2014.10.036)
Supplement: Supplementary data — This document contains supplementary figures and tables. [file mmc1.docx]

# Supplementary Material

**Supplementary Figure 1:** Singular value decomposition (SVD). An example output from implementing champ.SVD to reveal confounding variables within the matrix of beta values. This figure illustrates that there were negligible technical confounders associated with this subset of TCGA COAD samples.


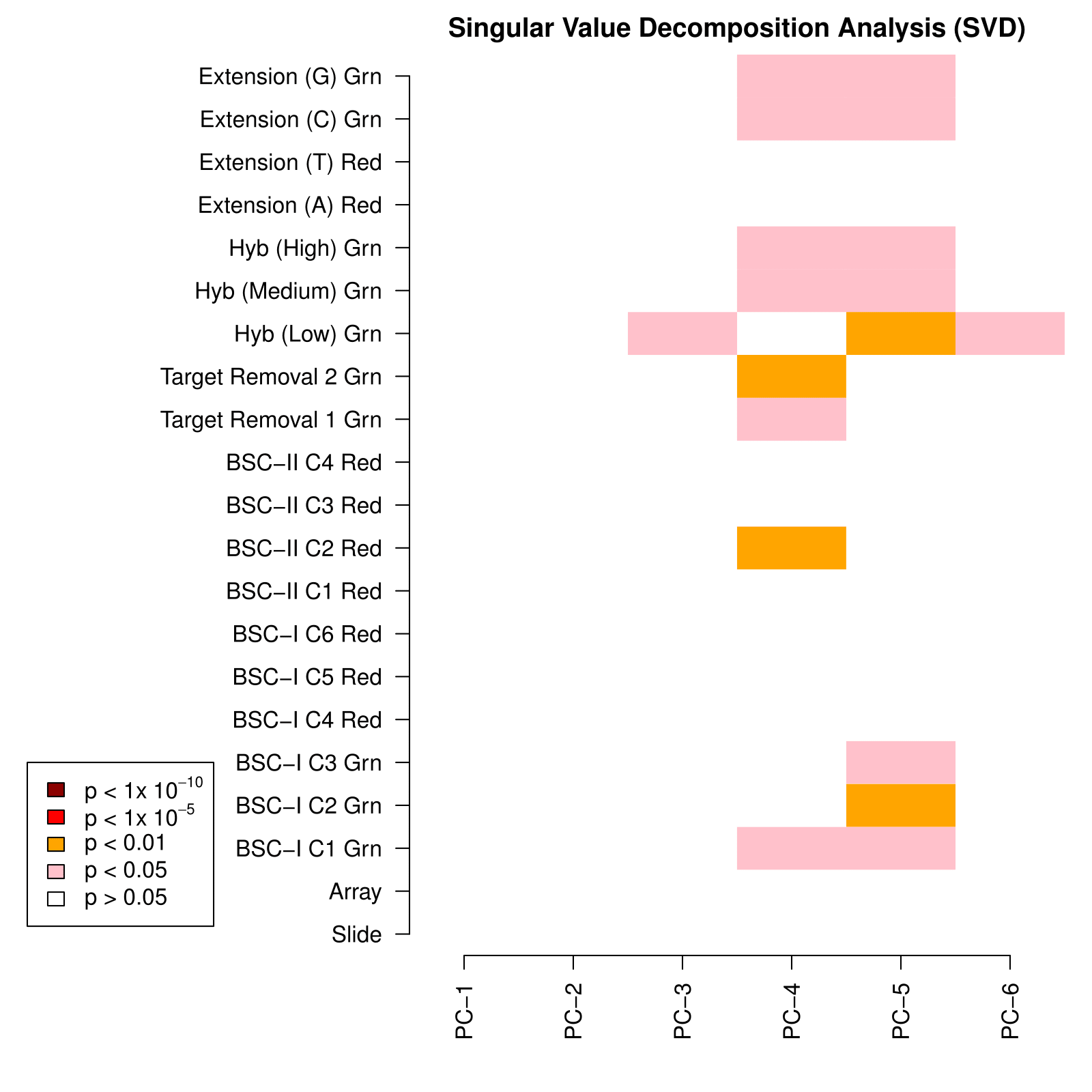


**Supplementary Figure 2:** Example output figure from Probe Lasso implemented in R using ChAMP illustrating the sizes of probe-lassos centred on probes in the various genetic/epigenetic features.

**
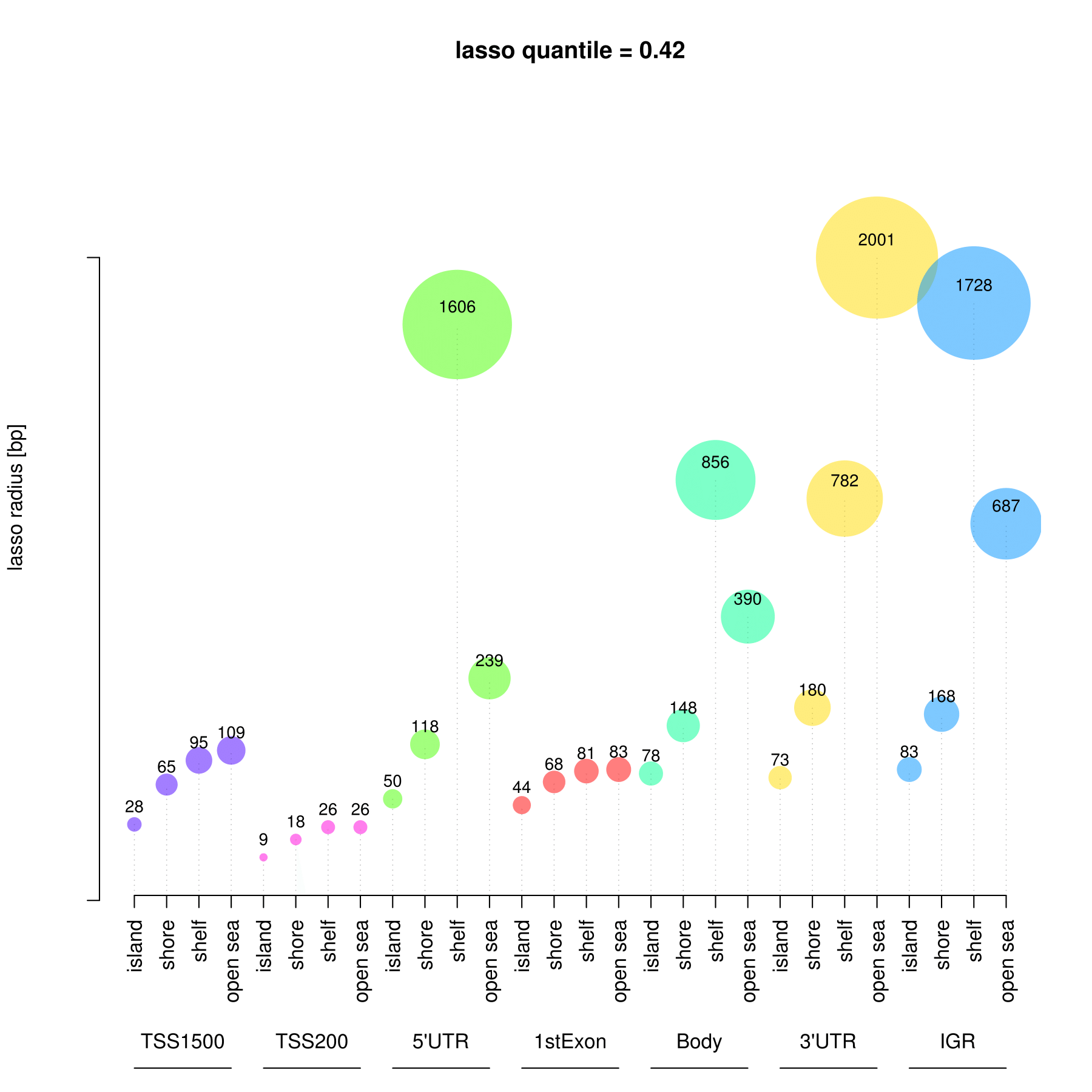
**

**Supplementary Figure 3**: Example output figure from Probe Lasso implemented in R using ChAMP illustrating the range of probe-spacing in the underlying dataset.

**
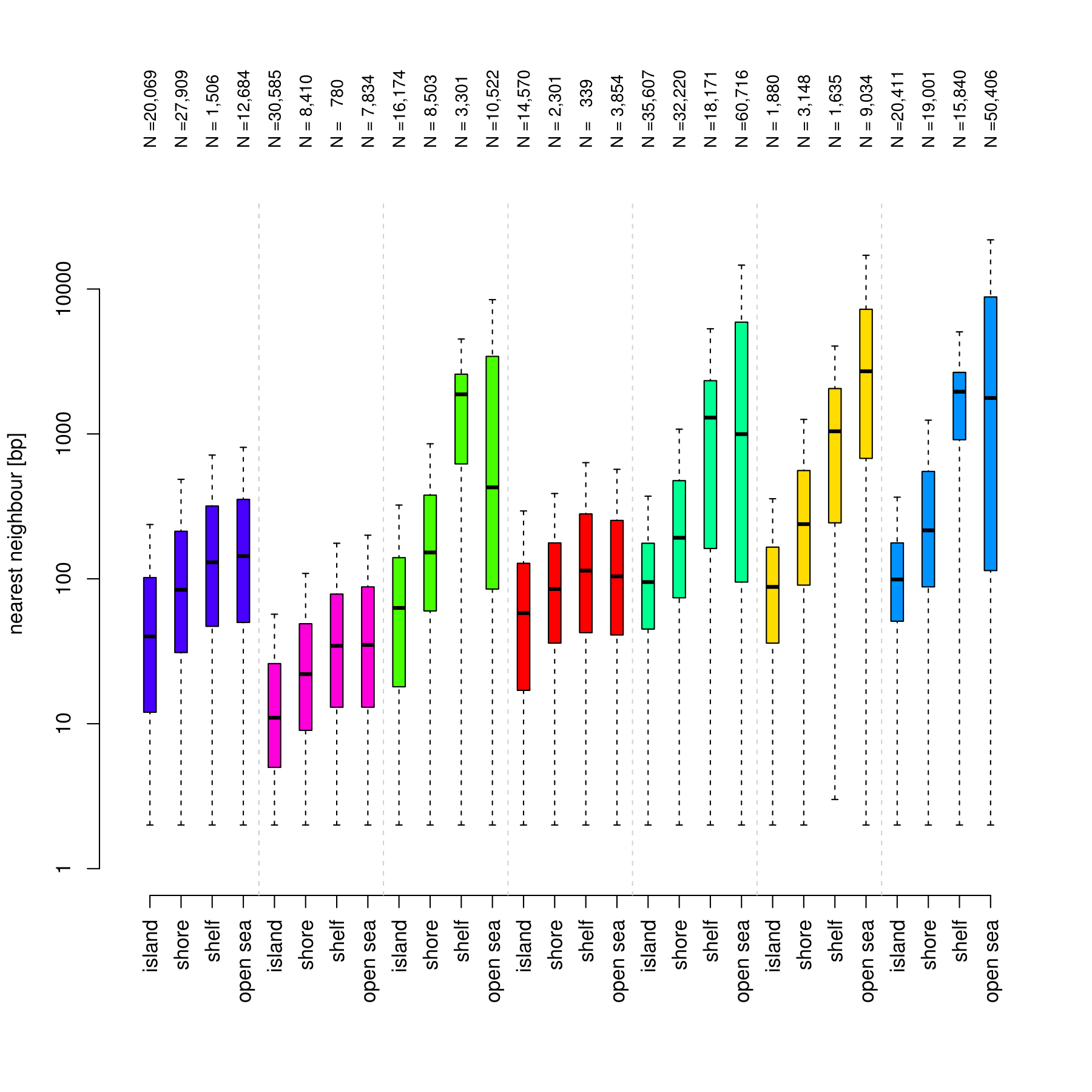
**

**Supplementary Figure 4**: Enrichment of gene relations (A) and CGI relations (B) for the lasso DMRs (dark grey) and window250 DMRs (mid greys) using stringency set at >= 3 significant probes captured. Enrichment is also presented using the distribution of significant MVPs only (light grey).


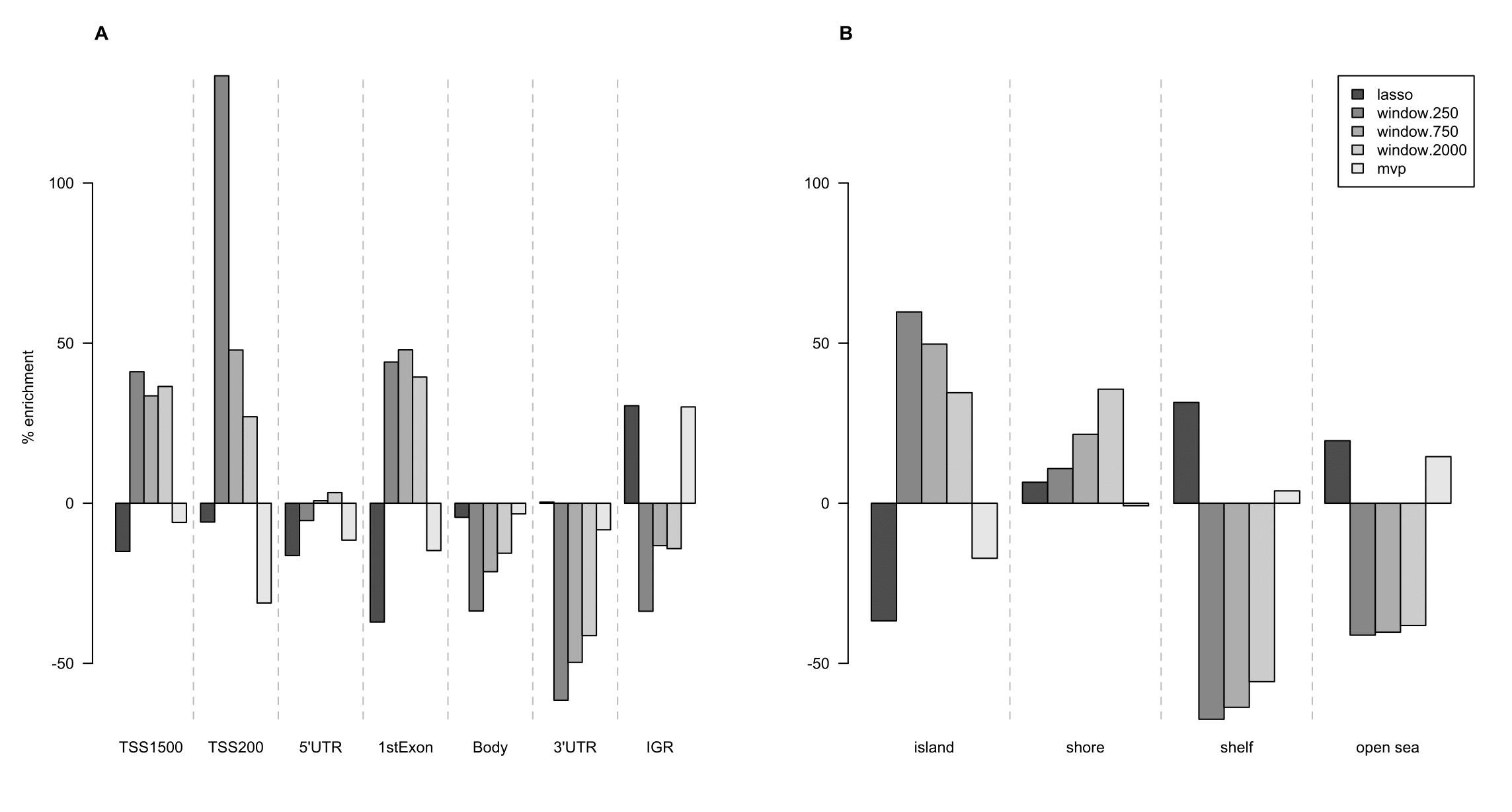


**Supplementary Figure 5:** Distributions of the sizes of DMR cores (space between first and last probes in a DMR) that result from the Probe Lasso (blue) and Sliding fixed window250 (red), window750 (green) and window2000 approaches. The results are split by stringency, with the effect being a gradual reduction in DMR size.


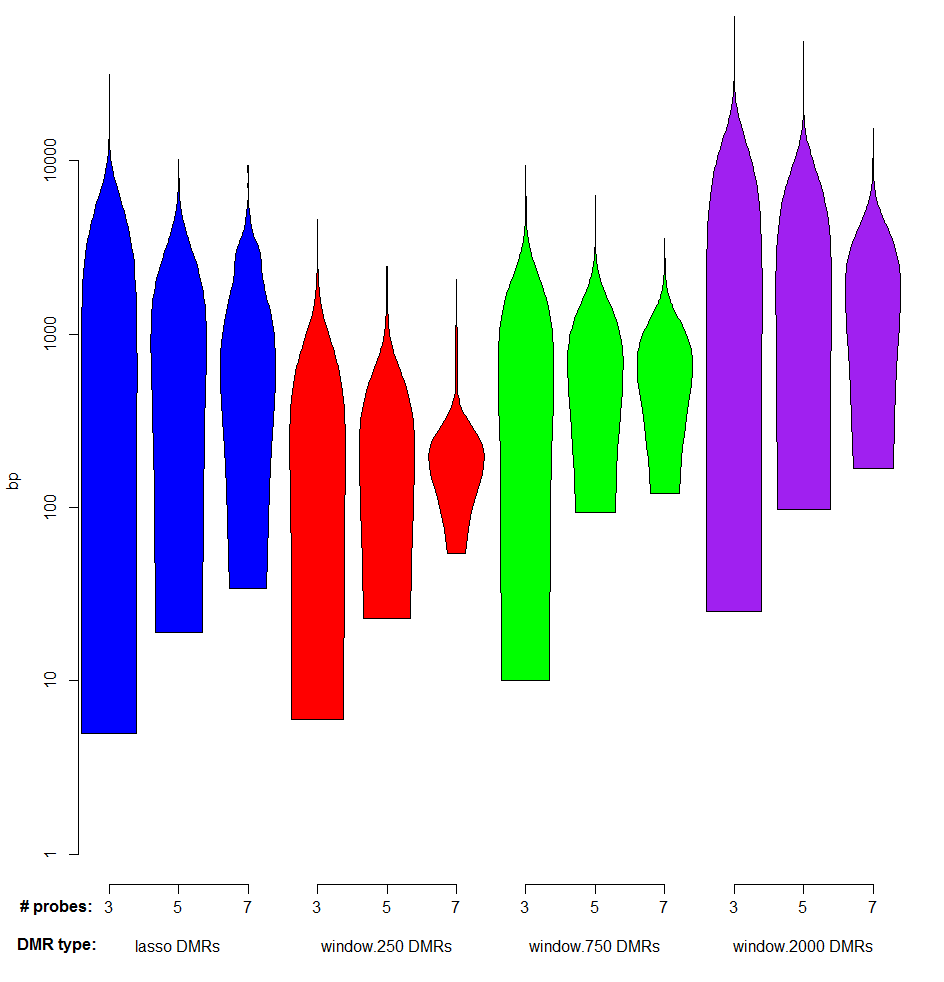


**Supplementary Figure 6:** Box plots to indicate the delta-beta between TCGA colorectal samples and healthy control colon for significantly associated probes that overlap with motifs enriched in unique DMRs. Negative delta beta means hypomethylation in colon cancer and vice-versa. Probes at enriched motifs in unique Probe Lasso DMRs were substantially hypomethylated (A) while probes at enriched motifs in unique window250 DMRs were considerably hypermethylated (B). The red vertical line indicates no methylation difference between cohorts.

**(A)**

**
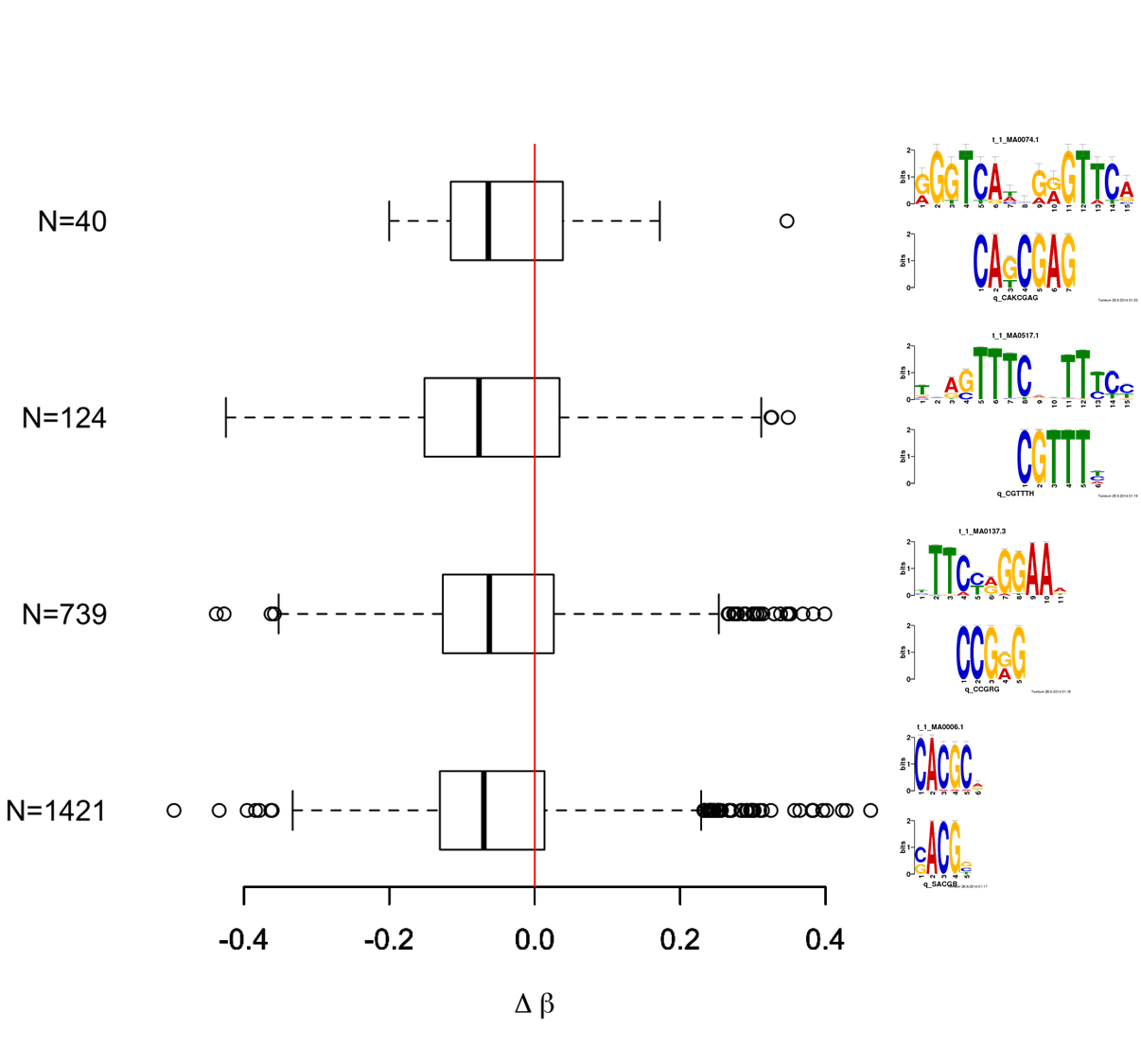
**

**(B)**

**
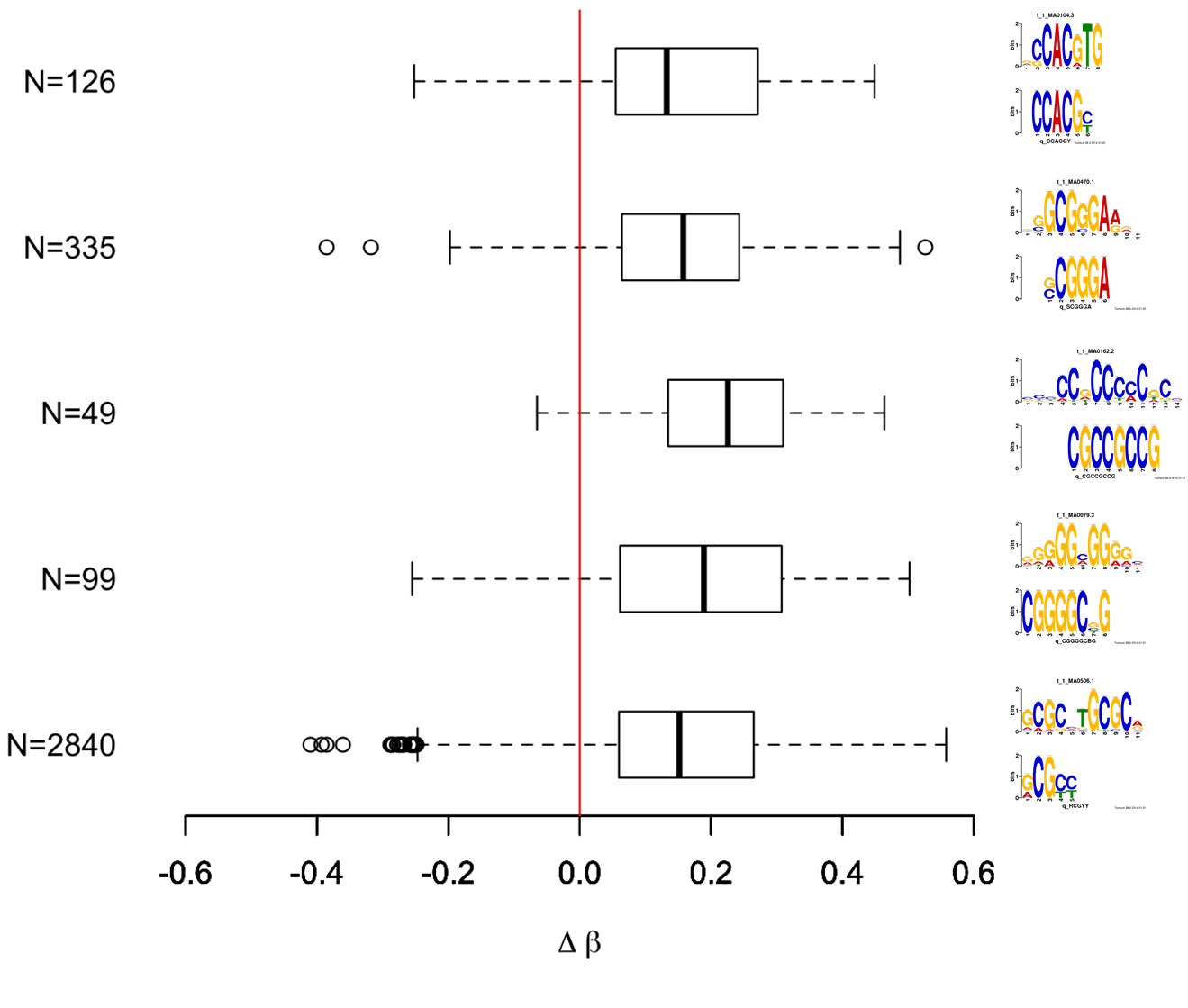
**

**Supplementary Table 1:** Identification of putatively DNA binding proteins associated with probes in DMRs unique to either Probe Lasso or window250 algorithms. Superscripts refer to the motif presented in Supplementary Figure 6 (1-5 = bottom-top).

| **motif match** | **Probe Lasso** | **Window250** |
| --- | --- | --- |
| Arnt | yes^1^ | yes^5^ |
| Arnt::Ahr | yes^1^ | yes^5^ |
| CTCF |  | yes^1^ |
| E2F1 |  | yes^2,4^ |
| E2F3 |  | yes^2,3,4^ |
| E2F4 |  | yes^2,4^ |
| E2F6 |  | yes^2,4^ |
| EGR1 |  | yes^2,3,5^ |
| EGR2 | yes^1^ | yes^5^ |
| EHF | yes^4^ |  |
| ELK4 |  | yes^4^ |
| GABPA |  | yes^4^ |
| HIF1A::ARNT | yes^1^ |  |
| INSM1 |  | yes^3^ |
| Klf1 |  | yes^5^ |
| Klf4 |  | yes^2,5^ |
| KLF5 |  | yes^2,3^ |
| Mafb | yes^1^ | yes^3^ |
| Myc |  | yes^5^ |
| MYC::MAX |  | yes^5^ |
| Mycn | yes^1^ | yes^5^ |
| NHLH1 |  | yes^3^ |
| NRF1 |  | yes^1,3^ |
| Pax2 | yes^1^ |  |
| PAX5 | yes^1^ |  |
| Pax6 | yes^1^ |  |
| PPARG | yes^1^ |  |
| REST |  | yes^1^ |
| RFX5 | yes^4^ |  |
| RXR::RAR_DR5 | yes^4^ |  |
| RXRA::VDR | yes^4^ |  |
| SP1 |  | yes^2,3^ |
| SP2 |  | yes^2,3,4^ |
| STAT1 | yes^2^ | yes^4^ |
| STAT2::STAT1 | yes^3^ |  |
| STAT3 | yes^2^ | yes^4^ |
| Stat4 | yes^2,3^ | yes^4^ |
| TAL1::GATA1 | yes^4^ |  |
| USF1 |  | yes^5^ |
| YY1 |  | yes^3^ |
| Zfx | yes^2^ | yes^2^ |

**Supplementary Table 2:** Gene ontology predictions for motifs overlapping with significantly associated probes in DMRs unique to Probe Lasso and window250 algorithms. Superscripts refer to the motif presented in Supplementary Figure 6 (1-5 = bottom-top). MF = Molecular function; CC = Cellular component; BP = Biological process.

| **category** | **GO prediction** | **Probe Lasso** | **Window250** |
| --- | --- | --- | --- |
| MF | zinc ion binding | yes^2^ |  |
| MF | transcription factor activity | yes^1^ |  |
| MF | transcription activator activity | yes^2^ | yes^3,5^ |
| MF | sequence-specific DNA binding | yes^1^ |  |
| MF | RNA binding | yes^3^ | yes^4^ |
| MF | protein serine/threonine kinase activity | yes^4^ |  |
| MF | magnesium ion binding |  | yes^1,3,5^ |
| MF | DNA binding | yes^3^ |  |
| MF | ATP binding | yes^2,4^ | yes^1,2,3,4,5^ |
| CC | transcription factor complex | yes^2^ | yes^2,4^ |
| CC | spliceosomal complex | yes^3^ |  |
| CC | nucleus | yes^1^ |  |
| CC | nucleolus | yes^3,4^ | yes^1,4,5^ |
| CC | microtubule |  | yes^3^ |
| CC | cytosol | yes^2^ | yes^2,3,4,5^ |
| BP | small GTPase mediated signal transduction |  | yes^2^ |
| BP | response to organic cyclic substance | yes^1^ |  |
| BP | regulation of transcription |  | yes^1^ |
| BP | protein folding |  | yes^1^ |
| BP | nuclear mRNA splicing, via spliceosome | yes^3,4^ |  |
| BP | negative regulation of cell migration | yes^1^ |  |
| BP | modification-dependent protein catabolic process |  | yes^2^ |
| BP | cell division | yes^4^ |  |
